# Longitudinal Surveillance of Influenza A Virus Exposure in Wild Boars ( *Sus scrofa* ) in Spain (2015–2023): Serologic and Virologic Evidence of Subtype Infections and H5N1 Spillover Risk

**DOI:** 10.1111/zph.70040

**Published:** 2026-02-10

**Authors:** Paloma Encinas, Aitor Nogales, Estela Escribano‐Romero, M. Ángeles Martín del Burgo, Jorge Ramón López‐Olvera, José Enrique Granados, Gregorio Mentaberre, Adolfo García‐Sastre, Gustavo del Real

**Affiliations:** ^1^ Department of Biotechnology National Institute of Agricultural and Food Research and Technology (INIA‐CSIC) Madrid Spain; ^2^ Department of Animal Health. Faculty of Veterinary Medicine Complutense University of Madrid Madrid Spain; ^3^ Center for Animal Health Research, (CISA, INIA‐CSIC) Madrid Spain; ^4^ Servei D'ecopatologia de Fauna Salvatge (SEFaS) and Wildlife Ecology & Health Group (WE&H), Departament de Medicina i Cirurgia Animals Universitat Autònoma de Barcelona Barcelona Spain; ^5^ Research Group RNM118 (Especies cinegéticas y Plagas) and Delegación Territorial de Sostenibilidad y Medio Ambiente en Granada Granada Spain; ^6^ Wildlife Ecology & Health Group (WE&H) and Departament de Ciència Animal Universitat de Lleida (UdL) Lleida Spain; ^7^ Department of Microbiology Icahn School of Medicine at Mount Sinai New York New York USA; ^8^ Global Health and Emerging Pathogens Institute Icahn School of Medicine at Mount Sinai New York New York USA; ^9^ Department of Medicine, Division of Infectious Diseases Icahn School of Medicine at Mount Sinai New York New York USA; ^10^ The Tisch Cancer Institute Icahn School of Medicine at Mount Sinai New York New York USA; ^11^ Department of Pathology, Molecular and Cell‐Based Medicine Icahn School of Medicine at Mount Sinai New York New York USA; ^12^ The Icahn Genomics Institute Icahn School of Medicine at Mount Sinai New York New York USA

**Keywords:** H5N1, high pathogenicity avian influenza, Influenza A virus (IAV), swine influenza, wild boar

## Abstract

**Introduction:**

Influenza A viruses (IAVs) are responsible for respiratory infections in a wide range of species, including birds, swine and humans. The role of wild boar ( *Sus scrofa* ) in IAV epidemiology remains underexplored. Here, we present a longitudinal serologic and virologic surveillance study of wild boars in Spain from 2015 to 2023.

**Methods:**

A total of 1643 nasal exudates and 2932 serum samples were analysed using quantitative RT‐PCR, ELISA and haemagglutination inhibition (HI) assays to detect IAV positive samples and IAV targeted antibodies to characterise circulating viral subtypes. In addition, in the context of highly pathogenic avian influenza H5N1 outbreaks, we explored the potential transmission of avian IAV to wild boar.

**Results:**

In summary, 6% of the serum samples tested positive and one IAV H3N1 was isolated. The seroprevalence remained stable from 2015 to 2018, undetected in 2019 and increased significantly from 2020 to 2023. The most frequently detected subtype was Eurasian avian‐like H1 (clade 1C) while pandemic H1 (clade 1A) and human‐like H1 (clade 1B) were less common. Human seasonal‐like H3 strains from the 2000s (2000s‐like H3) emerged in 2017 and have become more seroprevalent in recent years. A subset of wild boar sera from areas overlapping with H5N1 HPAI outbreaks in poultry and wild birds tested positive for recombinant H5 by ELISA, although H5N1 HI assays were negative.

**Conclusions:**

The monitoring of IAV in wild boar population allowed the identification of the temporal and spatial trends and shifts in the prevalence and characterisation of the infecting IAV strains. Our data suggest potential spillover events from human or other sources and support the inclusion of integrated monitoring of the wild suids as IAV reassortment‐prone hosts in influenza surveillance programs.

## Impacts

- This study shows that wild boars are continuously exposed to a variety of IAV subtypes from different clades. However, we cannot determine whether some of these exposures result in transmission of IAV among boars or to other susceptible species; the coinfection with two distinct strains in wild boars may produce novel IAVs by reassortment. The growing, expanding and increasingly synurbic wild boar population is likely to result in greater and closer interactions with livestock, wildlife and humans, favouring interspecies transmission of IAV.
- Although no serological evidence of H5N1 was found in wild boar in this study, positive serology has already been detected in Europe and avian influenza epidemic events continue to occur in Spain, increasing the risk of the possible emergence of mutations that enable avian viral strains to adapt to pigs, favouring pig‐to‐pig transmission, as it has already happened in minks and cows.
- Targeted IAV surveillance in wild boar as part of integrated wildlife monitoring, sympatric avian populations and humans both in rural and urban environments is necessary to monitor the IAV strains circulating in nature and forecast potential IAV reassortment and/or mutations that could trigger outbreaks in populations and species newly susceptible to infection and intraspecies transmission.

## 1 Introduction

Influenza A virus (IAV) is a member of the *Orthomixiviridae* family and it is classified into subtypes based on the two main glycoproteins located on the surface of the virus: the haemagglutinin (HA) and the neuraminidase (NA). It is the causative agent of influenza A, an infectious disease affecting a wide range of hosts: birds and mammals, including humans. IAV causes a high impact in the pig production industry as one of the viral factors involved in the development of porcine respiratory disease complex (PRDC) (Lagan et al. 2024), and causes relevant economic losses and variable mortality depending on strain virulence and population pre‐existing immunity (Krammer et al. 2018). In addition, sporadic human influenza pandemic events involving IAV strains of zoonotic origin, mainly avian and porcine, have occurred (Krammer et al. 2018). An unprecedented epidemic wave of avian H5N1 IAV affecting wild and captive birds worldwide is currently raising great health public concern as it is affecting not previously described hosts, such as cows in the United States (Peacock et al. 2025) through not completely understood transmission ways (Perez‐Acle et al. 2024). European authorities and wildlife associations strongly recommend expanding compulsory surveillance of avian IAV to carnivores and scavenger mammals, as there are still many gaps in the knowledge about this strain and the situation is rapidly evolving (EFSA et al. 2023; EFSA Panel on Animal Health et al. 2025).

Swine populations play an important role in the ecology of avian influenza viruses (AIVs) as they carry viral receptors for both avian and human origin IAVs in their respiratory tract (Ma et al. 2023). In fact, reverse zoonoses from humans to pigs, are common worldwide (Trovao and Nelson 2020). Commonly, swine influenza virus (SIV) strains from diverse origins, clades and subclades converge and reassort in domestic pigs and wild boars, both belonging to the same specie (*Sus scrofa*), producing new SIVs that can be maintained enzootically while undergoing antigenic drift (Goel et al. 2025; Richard et al. 2025). In swine, H1N1, H1N2, H3N2 and H3N1 subtypes have been described in Spain (Encinas et al. 2022), with H1 haemagglutinin from Eurasian avian‐like (EAswH1), human seasonal‐like (HUswH1) and pandemic‐like (PDMswH1) lineages, as well as human seasonal‐like H3 from 1970s (1970s‐like H3) and novel H3 from 2000s (2000s‐like H3) (Anderson et al. 2021). AIVs present the most diverse group of HA/NA combinations and can be divided into highly pathogenic avian influenza (HPAI) and low pathogenic avian influenza (LPAI) strains. The HPAI viruses primarily affect birds, with the H5N1 2.3.4.4.b clade being the most common in wild birds and poultry since 2020. HPAI H5N1 clade 2.3.4.4.b outbreaks have been detected in wild birds, poultry (Ministerio de Agricultura, Pesca y Alimentación 2025a) and mammals in Europe and worldwide (Van Leeuw et al. 2025). An HPAI outbreak was reported in a fur farm in Spain in 2022, with sustained transmission in American minks ( *Neovison vison* ) and presence of genomic markers of adaptation to mammals (Agüero et al. 2023). Pigs are also susceptible to avian‐derived HPAI H5N1 (United States Department of Agriculture (USDA) 2024). Serological detection of antibodies against HPAI H5N8 viruses in wild boars in Germany (Schülein et al. 2021) and against HPAI H5N1 in pigs from a free range multispecies rural farm in France (Herve et al. 2021) have provided new insights into the potential interspecies transmission routes and raise the hypothesis that wild suids may be exposed to H5N1 under natural conditions.

The wildlife‐livestock interface appears to be the ideal scenario for the exchange and spread of infectious diseases of zoonotic concern (Wiethoelter et al. 2015). Free‐ranging wild boars frequently interact with wildlife and livestock, including open‐air raised domestic Iberian black pigs, especially in extensive production systems (Cadenas‐Fernández et al. 2019; Triguero‐Ocaña et al. 2021). In recent decades, wild boar populations have expanded worldwide and increasingly colonised urban areas across Europe in search of new food sources (Castillo‐Contreras et al. 2021; Conejero et al. 2024). This synurbization may increase the risk of pathogen spillover at the human‐wildlife interface, making wild boars a relevant sentinel species for zoonotic disease surveillance (Barroso et al. 2023). Despite their ecological relevance, the role of wild boar populations in the transmission and maintenance of IAVs remains poorly explored compared to domestic swine. Epidemiological studies across Europe have consistently reported evidence of IAV circulation in wild boars, although the seroprevalence rates and the circulating subtypes vary significantly among regions (De Marco et al. 2022; Delogu et al. 2019; Grech‐Angelini et al. 2018; Kovalenko et al. 2017; Malmsten et al. 2018; Markowska‐Daniel et al. 2015; Prosperi et al. 2022).

In this study, we aim to evaluate the potential contribution of wild boar to IAV ecology in Spain by estimating annual seroprevalence over a nine‐year period (2015–2023) and identifying the main subtypes circulating in this population. Special attention was given to the potential transmission of H5N1, most likely from avian hosts, to wild boars following the occurrence of HPAI outbreaks in overlapping habitats.

## 2 Materials and Methods

### 2.1 Study Area and Biological Samples

**FIGURE 1:**
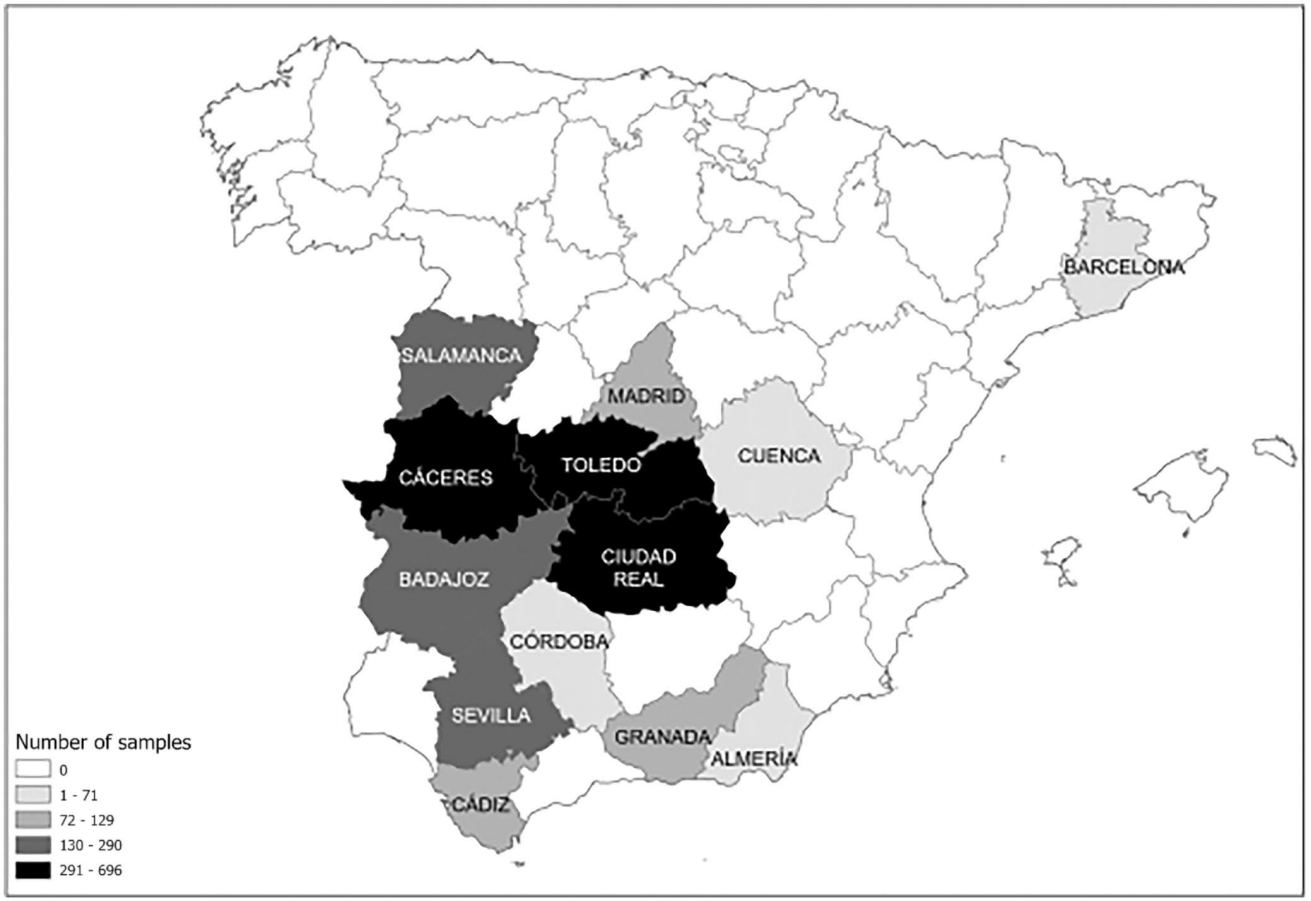
Spatial distribution of the samples analysed during 2015–2023 period.

A total of 2909 blood and 1643 nasal exudate samples were opportunistically collected post‐mortem from hunted or captured and euthanized wild boars across Spain between 2015 and 2023 during routinary surveillance campaigns. Most of the samples originated from rural areas of 12 provinces belonging to 4 regions in south‐central Spain: Salamanca, Madrid, Toledo, Cuenca, Ciudad Real, Cáceres, Badajoz, Almería, Cádiz, Granada, Córdoba and Sevilla (Figure 1). In addition, 23 blood samples were obtained in 2017 from synurbic wild boars from the Metropolitan Area of Barcelona (MAB) in northeastern Spain. Blood samples were collected from the cavernous or orbital sinus (Arenas‐Montes et al. 2013) and processed by centrifugation at 1200 *g* for 10 min to separate the sera, which were then stored at −20°C until further analysis. A specific subset of sera was selected based on spatio‐temporal overlap with HPAI H5N1 outbreaks in poultry or wild birds, as documented through the national animal health passive surveillance program between late 2021 and early 2023 (Ministerio de Agricultura, Pesca y Alimentación 2024a). The nasal exudate samples were obtained by inserting sterile cotton swabs into the ventral part of the nose and gently rotating them for a few seconds around the nasal cavity. The moistened swabs were then inserted into viral transport medium (VTM), a phosphate buffer solution containing 0.5% bovine serum albumin (BSA), 200 U/mL penicillin, 200 mg/mL streptomycin, 100 U/mL polymyxin B sulphate and 250 mg/mL gentamicin. The swabs were chilled and sent to the laboratory within 24–36 h after collection, and if this was not possible, they were stored at −80°C until use.

### 2.2 Serological Assays

**FIGURE 2:**
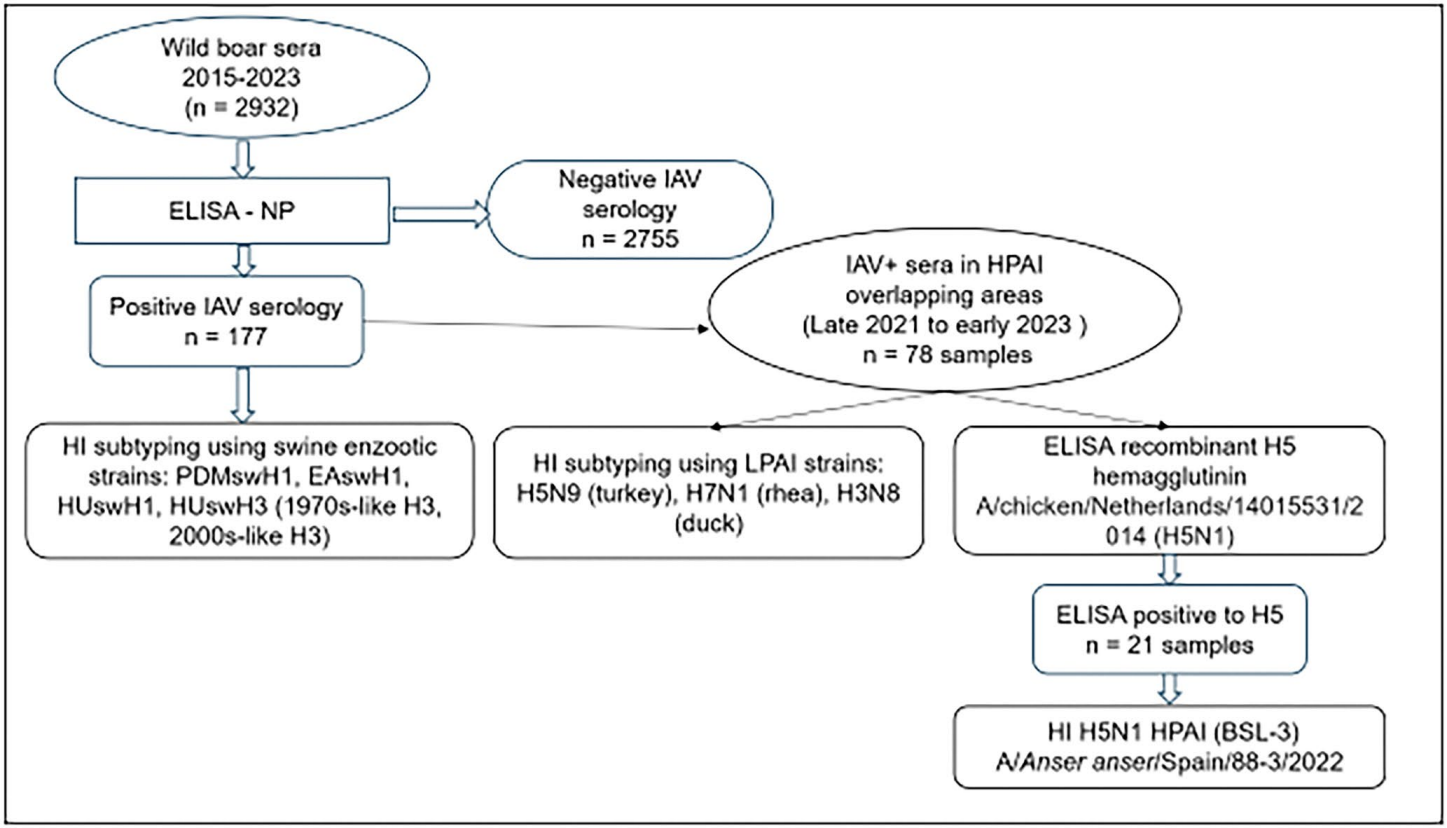
Schematic workflow of the wild boar sera processing and influenza A serological testing. +, positive; BSL‐3, biosafety level 3 conditions; EAswH1, Eurasian avian‐like HA; ELISA, enzyme‐linked immunosorbent assay; H1, haemagglutinin 1; HI, haemagglutination inhibition; HPAI, high pathogenic avian influenza; HUswH1, human seasonal‐like H1; HUswH3, human seasonal‐like haemagglutinin H3 introduced in 1970 or 2000 in European swine populations; IAV, Influenza A virus; LPAI, low pathogenic avian influenza; NP, Influenza A nucleoprotein; PDMswH1, human pandemic‐like H1.

The wild boar serum samples were processed following the workflow illustrated in Figure 2. Initially, all the sera were screened for antibodies against IAV using a commercial indirect enzyme‐linked immunosorbent assay (ELISA) targeting the viral nucleoprotein (NP), according to the manufacturer's instructions (INGEZIM Influenza A, Eurofins‐Ingenasa, Spain).

Positive sera were subsequently analysed by haemagglutination inhibition (HI) assay using reference SIV antigens. The HI assay was conducted according to the standard procedures established by the World Health Organization (WHO) (World Health Organisation 2011). Prior to testing, the ELISA‐positive sera were treated overnight (O/N) with receptor‐destroying enzyme (RDE) from *Vibrio cholerae* (Sigma, Spain) to remove nonspecific inhibitors, followed by inactivation at 56°C for 60 min. Subsequently, the sera were adsorbed with turkey red blood cells (RBCs) at 4°C to prevent nonspecific agglutination. The cutoff of HI was set to dilution ≥ 1:20.

The SIV strains used in the HI assays reflect the SIV genetic lineages previously described in Spain: Eurasian avian‐like H1 (EAswH1) clade 1C, human seasonal‐like H1 (HUswH1) clade 1B, pandemic‐like H1 (PDMswH1) clade 1A and human seasonal‐like H3 (HUswH3) from the 1970s (1970s‐like H3) and novel human seasonal‐like H3 from the 2000s (2000s‐like H3) (Encinas et al. 2022). All the SIV strains used in this study come from Spanish isolates propagated in Madine‐Darby canine kidney (MDCK) cells, and LPAI strains have been propagated in chicken embryos. SIV strains within the same clade have been updated to obtain antigens as broadly cross reactive as possible with the specific subtype according to the World Organisation for Animal Health (WOAH) (2024) guidelines. Clade assignment followed the Global Swine Nomenclature System (Anderson et al. 2016) as implemented through the classification tool of the Bacterial and viral bioinformatics resource centre (Olson et al. 2023). Full details of the viral strains used are available in Table 1. Sera from domestic pigs with a defined HI titre against the respective antigens were used as controls. If the sera showed positive HI titres to two or more antigens, the one with a four or more‐fold difference with the others was considered positive. If positive HI titres were found to different antigens and they had similar magnitudes, the sample was considered positive to both antigens. All the sera samples considered IAV‐positive, as previously noted, but not clearly reactive in HI were described as ‘undertermined subtype’.

**TABLE 1:** Description of the viral strains used in this study.

Name	Subtype	Lineage	Clade	Accession number
A/California/07/09	H1N1	PDMswH1	1A.3.3.2	NC_026433.1
A/swine/Spain/45690–9/2018	H1N2	PDMswH1	1A.3.3.2	MZ945846.1
A/swine/Spain/53207/2004	H1N1	EAswH1	1C.2.1	KR700597.1
A/swine/Spain/45700–1/2016	H1N2	EAswH1	1C.2.1	MF872838.1
A/swine/Spain/40250–1/2016	H1N1	EAswH1	1C.2.1	MF872846.1
A/swine/Spain/6370–1/2018	H1N2	EAswH1	1C.2.1	PQ047764.1
A/swine/Spain/45534–1/2019	H1N2	EAswH1	1C.2.1	MZ945771.1
A/swine/Spain/40564/2002	H1N2	HUswH1	1B.1.2	CY116550.1
A/swine/Spain/45600–1/2017	H1N2	HUswH1	1B.1.2	MZ945809.1
A/swine/Spain/50001–1/2019	H1N2	HUswH1	1B.1.2	MZ945794.1
A/swine/Spain/54008/2004	H3N2	HUswH3	1970.1	CY010564.1
A/swine/Spain/45690–1/2016	H3N2	HUswH3	1970.1	MF872855.1
A/swine/Spain/45690–10/2018	H3N1	HUswH3	2000.3	MZ945763.1
A/swine/Spain/31001–1/2019	H3N1	HUswH3	2000.3	MZ373162.1
A/wild boar/Spain/45560–1/2021	H3N1	HUswH3	2000.3	PP338808.1
A/turkey/Wisconsin/1/1968	H5N9	LPAI		CY080507
A/rhea/North Carolina/39482/1993	H7N1	LPAI		EF470586
A/duck/UKR/1/1963	H3N8	LPAI		CY006038
A/chicken/Netherlands/14015531/2014	H5N8	HPAI	2.3.4.4c	EPI_ISL_18600237 ¥
A/ *Anser anser* /Spain/88–3/2022	H5N1	HPAI	2.3.4.4b	OM943965.1

*Note:* Lineage was assigned following the global swine influenza classification (Anderson et al. 2016). Most accession numbers are from Genbank at the National Centre of Biotechnology Information (NCBI), with the exception of ¥, which originates from the Global Initiative on Sharing All Influenza Data (GISAID) database.

Abbreviations: EAswH1, Eurasian avian‐like HA; HPAI, high pathogenic avian influenza; HUswH1, human seasonal‐like H1; HUswH3, human seasonal‐like haemagglutinin H3 introduced in 1970s or 2000s in European swine populations; LPAI, low pathogenic avian influenza; PDMswH1, pandemic‐like H1.

To assess the potential exposure of wild boars to AIV, a subset of 78 sera was further tested by HI against LPAI strains: H5N9 (turkey), H7N1 (rhea) and H3N8 (duck), using the same protocol applied with the swine strains. In addition, a second ELISA using a recombinant haemagglutinin protein (Meade et al. 2017) derived from the AIV strain A/chicken/Netherlands/14015531/2014 (H5N1) was performed to determine H5‐specific antibodies. Briefly, ELISA plates (Polysorp, Nunc) were coated with 100 ng of recombinant H5 protein and incubated O/N at 4°C. The plates were washed twice with phosphate‐buffered saline (PBS) containing 0.05% Tween‐20 (PBST) and then blocked for 1 h at room temperature (RT) with PBS containing 2% bovine serum albumin (BSA). After 3 washes, wild boar sera diluted 1:600 were added and incubated for 1 h at 37°C. Plates were washed again and incubated with goat anti‐pig horseradish peroxidase (HRP)‐conjugated antibody (1:10,000 in PBS‐BSA 0.5%) for 30 min at RT. After 5 washes, o‐phenylenediamine dihydrochloride (OPD) substrate was added, and reactions were stopped after 10 min with 3 M HCl. The optical density at 492 nm was measured. The positive controls included sera from mice immunised with LPAI (A/turkey/Wisconsin/1/1968, H5N9) and appropriate secondary antibodies. The negative controls included irrelevant immunised mice sera against AIV (A/rhea/North Carolina/39482/1993, H7N1). The cutoff was determined as the mean absorbance of negative controls plus two standard deviations.

Finally, the recombinant H5‐positive sera were further analysed by HI assay using a contemporary HPAI strain, isolated in recent outbreaks in Spain: A/ *Anser anser* /Spain/88–3/2022 (H5N1) under biosafety level 3 (BSL‐3) conditions at the Centre for Animal Health (CISA, INIA‐CSIC).

### 2.3 RNA Extraction and Virus Isolation

Upon receipt at the laboratory, the nasal swabs were vigorously agitated using a vortex mixer and centrifuged at 1900 *g* for 15 min at 4°C and the supernatants were used for total RNA extraction, using the QIAamp Viral RNA Mini Kit (Qiagen) in a QIACube Station (Qiagen). IAV detection was made by real time reverse transcription quantitative polymerase chain reaction (RT‐qPCR) of the IAV matrix (M) gene with the following primers. Forward: 5′‐GACCRATCCTGTCACCTCTGAC, reverse: 5′‐AGGGCATTYTGGACAAAKCGTCTA, probe: TGCAGTCCTCGTTCACTGGGCACG (labelled with 6‐FAM, MGBNFQ; Invitrogen). The reaction conditions were: 45°C/30′, 95°C/10′, 40× (95°C/15″, 60°C/60″). The RT‐qPCR was performed with the High Scriptools‐Quantimix Easy Probes Kit (Biotools) in an Applied Biosystems TM Fast 7500 thermal cycler. IAV isolation from the RT‐qPCR positive samples (Ct value < 35 cycles) was made in MDCK cell cultures grown in advanced Dulbecco's Modified Eagle Medium (DMEM) (Thermo Fisher) supplemented with glutamine (4 μM), 4‐(2‐hydroxyethyl)‐1‐piperazineethanesulfonic acid (HEPES) (20 μM), BSA fraction V, 100 U/mL streptomycin, 100 U/mL penicillin and TPCK (6‐(1‐tosylamido‐2‐phenyl)‐ethyl‐chloromethyl‐ketone)‐treated trypsin (2 mg/mL) from bovine pancreas (Merck Life Science) at 37°C and 5% CO_2_ or in 10‐day‐old chicken embryos according to the Manual on Animal Influenza Diagnosis and Surveillance (World Health Organisation 2011). Before inoculation in embryos and cells, the nasal fluids were filtered through 0.22‐mm Millex syringe filters (Millipore). The inoculated cell cultures were cultured at 37°C, 5% CO_2_, and examined daily for evidence of cytopathic effect (CPE). If CPE was positive, or after 3 days, the supernatants were tested by RT‐qPCR. Allantoic fluid was tested for the presence of virus by haemagglutination assay or RT‐qPCR, and only negative allantoic fluid and cell supernatants were passaged up to 3 times. Nevertheless, samples with a Ct value comprised between 35 and 40 were also inoculated in MDCK cells and chicken embryos. The virus isolates were submitted to genetic analyses to determine the subtype by conventional one‐step RT‐PCR with the Superscript III one‐step RT‐PCR Kit with Platinum Taq polymerase and Sanger sequencing prior to whole genome sequencing.

### 2.4 Whole Genome Sequencing

The entire genome of viral isolates was amplified from 5 μL of RNA template using a multisegmented RT‐PCR (M‐RT‐PCR) strategy (Zhou et al. 2009). The RNA was extracted using a QIAamp viral RNA minikit (Qiagen) and the M‐RT‐PCR amplification was performed with the SuperScript III high‐fidelity RT‐PCR kit (Invitrogen) according to the manufacturer's instructions using the Opti1 primer set, consisting of primers: Opti1‐F1 (5′‐GTTACGCGCCAGCAAAAGCAGG), Opti1‐F2 (5′‐GTTACGCGCCAGCGAAAGCAGG), and Opti1‐R1 (5′‐GTTACGCGCCAGTAGAAACAAGG). The DNA amplicons were purified using an Agencourt AMPure XP 5 mL kit (Beckman Coulter) and quantified using the Qubit dsDNA HS Assay (Invitrogen). Sequencing libraries were prepared using the Ligation Sequencing Kit SQK‐LSK110 (Oxford Nanopore Technology, ONT) and NEBNext Quick Ligation Module (New England Biolabs), and sequencing was performed in a Minion Device (Oxford Nanopore Technology, ONT) with a Flonge Expansion Module using a Flow cell (FLO‐FLG001). Base‐calling of raw sequence reads (fast5) was done using Guppy software (ONT) and extraction of consensus sequences was performed thanks to the Flu Module of the Iterative Refinement Meta‐Assembler (IRMA) (Shepard et al. 2016) using the computing resources of the Galicia Supercomputing Centre (CESGA). Alternatively, and for confirmation purposes, full genome sequencing was also performed at Icahn School of Medicine at Mount Sinai (ISMMS) using a MiSeq instrument (Illumina, Cambridge, UK) with 2 × 150‐base paired‐end reads. The sequences were deposited in the National Centre for Biotechnology Information's (NCBI) GenBank. The IAV genotyping was performed as described in previous studies (Encinas et al. 2022).

### 2.5 Statistical Analysis

Fisher's exact test was performed in GraphPad Prism 9 to test the wild boar population for statistically significant differences in IAV seroprevalences among years and provinces. The significance threshold was set at a *p*‐value < 0.05.

### 2.6 Geographical Distribution

Maps showing geographical distribution of samples were generated using ArcGIS Pro 3.3 (ESRI, Redlands, CA, USA).

### 2.7 Ethical Statement

No specific ethical approval was required for this study, as biological samples were collected from free‐living wild boars trapped and euthanised or shot by veterinarians and wildlife control operators within the regular management of the species in compliance with the National Management Strategy for controlling wild boar populations. During this study, all wild boars were handled and sampled in accordance with standardised procedures in veterinary practices. No wild boars were specifically hunted or captured for the purpose of this study.

## 3 Results

### 3.1 Serological Assays

**FIGURE 3:**
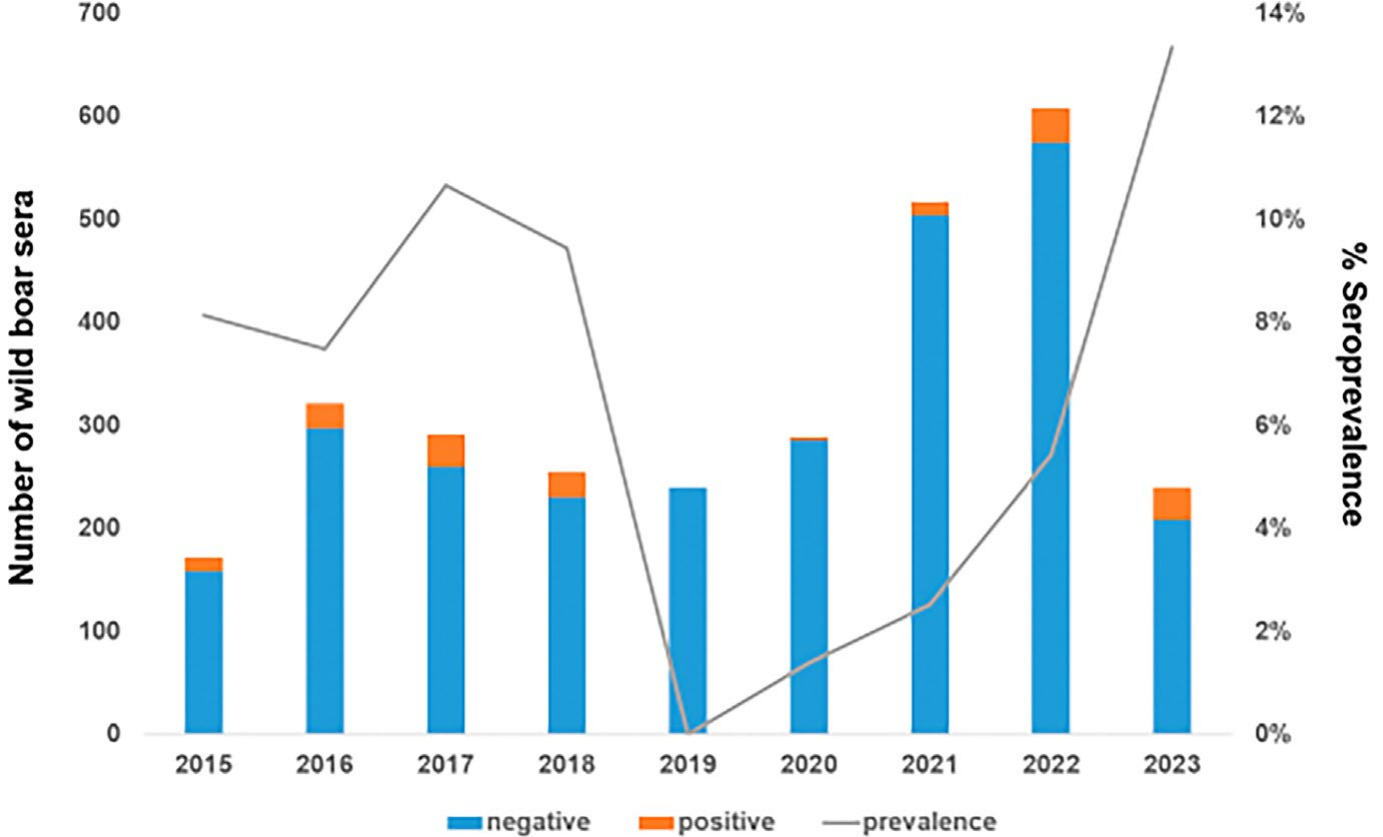
Influenza A screening of wild boar sera samples. Columns: Total number of sera per year; orange: Positive samples; blue: Negative samples. Grey line: Seroprevalence rate.

Out of the 2932 wild boar serum samples analysed, 177 tested positive for antibodies against IAV, resulting in an overall seroprevalence of 6%. The annual seroprevalence remained relatively stable between 2015 and 2018, with no statistically significant differences among these years. Conversely, no positive samples were detected in 2019. Afterwards, a significant upward trend in seroprevalence was observed (*p* < 0.05) from 2020 to 2023, in parallel with an increased sampling effort, detecting the highest seroprevalence in 2023 (Figure 3; Figure S1).

**TABLE 2:** Serological and virological analysis of influenza A virus (IAV) in wild boar population in Spain from 2015 to 2023.

Year	SP	IAV + SP	Serologic test (NP ELISA)				Virologic test (M RT‐qPCR)	
			Samples	IAV+	%	95% CI	Samples	IAV+
2015	12	2	172	14	8.14	4.05–12.23	37	0
2016	27	6	321	24	7.48	4.60–10.35	202	0
2017	37	8	291	31	10.65	7.11–14.20	20	0
2018	39	7	254	24	9.45	5.85–13.05	61	0
2019	32	0	231	0	0	**—**	258	0
2020	36	1	270	4	1.48	0.04–2.92	257	0
2021	51	5	515	13	2.52	1.17–3.88	266	1
2022	60	9	588	33	5.61	3.75–7.47	379	0
2023	24	7	240	32	13.33	9.03–17.63	161	0

*Note:* The seroprevalence trend followed a statistically significant increasing trend from 2019 to 2023. See additional information about *p*‐values among years in Figure S1. M RT‐qPCR: retro‐transcription quantitative PCR targeting IAV matrix gene using a FAM probe.

Abbreviations: %, seroprevalence percentage as number of positive cases/total number of samples per year; 95% CI, 95% central confidence interval; IAV+, positive; NP ELISA, enzyme‐linked immunosorbent assay targeting IAV nucleoprotein; SP, sampling points.

**FIGURE 4:**
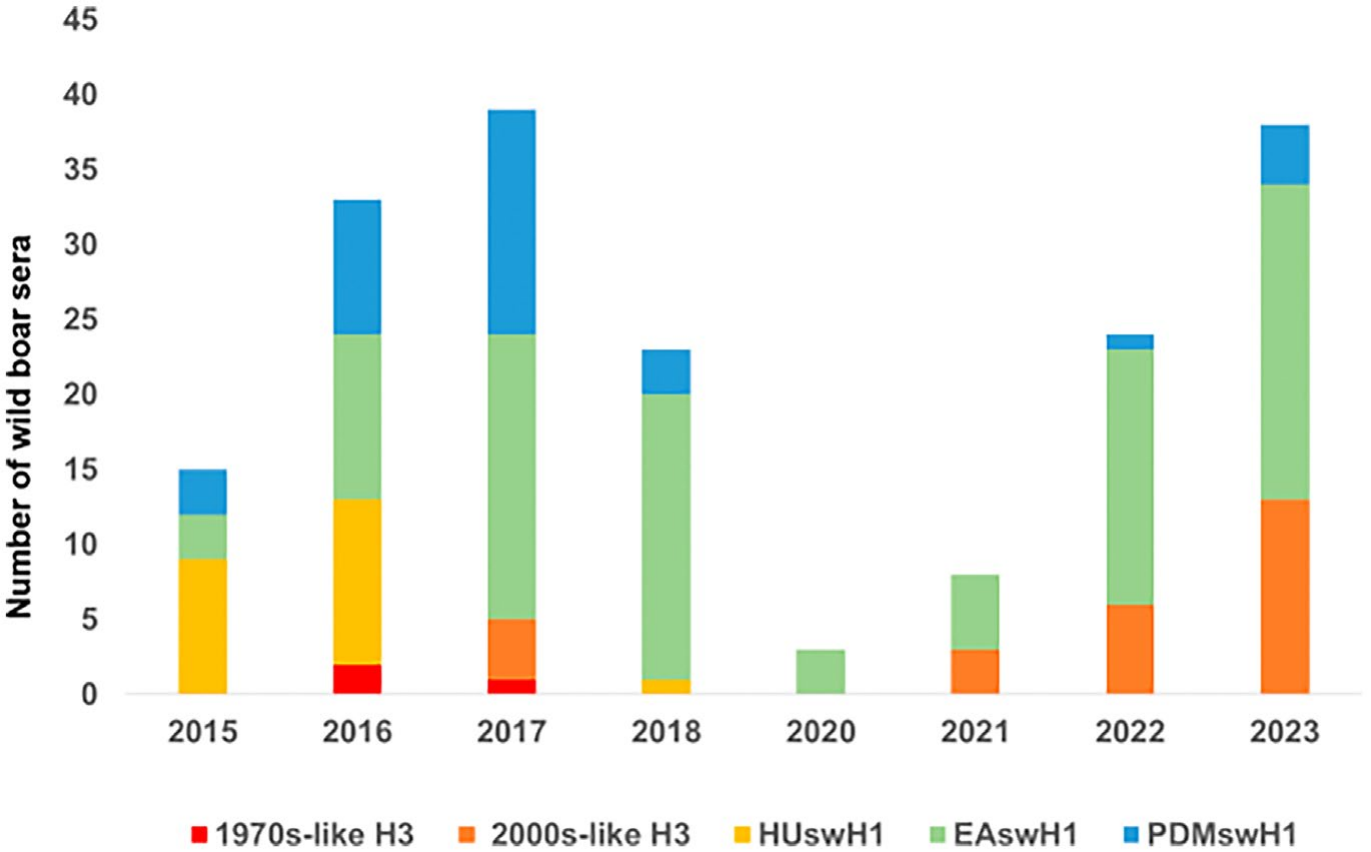
Interannual comparison of influenza A virus subtypes in wild boar sera. Column height represents number of counts of each subtype. Green, Eurasian avian‐like H1 (EAswH1,1C); yellow, Human seasonal‐like H1 (HUswH1, 1B); blue, pandemic‐like H1 (PDMswH1, 1A); red, 1970s‐like H3; orange, 2000s‐like H3.

Eurasian avian‐like H1 (EAswH1, clade 1C) was the most frequently detected subtype throughout the entire study period. The human seasonal‐like H1 (HUswH1, clade 1B) and pandemic‐like H1 (PDMswH1, clade 1A) lineages were prevalent between 2015 and 2018, with HUswH1 disappearing in the subsequent years. The 1970s‐like H3 lineage was detected in 2016 and 2017, whereas the 2000s‐like H3 lineage first emerged in 2017 and progressively became the second most detected subtype from 2021 onwards (Figure 4, Table 3).

**TABLE 3:** Subtype assignment of influenza A positive sera samples.

Year	HA lineage					
	EAswH1	PDMswH1	HUswH1	1970s‐like H3	2000s‐like H3	Undet
2015	3	3	9			
2016	11	9	11	2		3
2017	19	15		1	4	7
2018	19	3	1			7
2020	3					
2021	5				3	3
2022	17	1			6	9
2023	21	4			13	2

Abbreviations: 1970s‐like H3, H3N2 with a human seasonal‐like H3 haemagglutinin introduced in European swine population in 1970s; 2000s‐like H3, H3N1 subtype with a HA from clade 2000.3; EAswH1, H1N1 or H1N2 subtypes with a HA belonging to Eurasian avian‐like H1; HA, haemagglutinin; HUswH1, H1N2 subtype with a HA belonging to human seasonal‐like H1; PDMswH1, H1N1 or H1N2 subtypes with a HA belonging to pandemic‐like H1; Undet, no subtype assigned.

**FIGURE 5:**
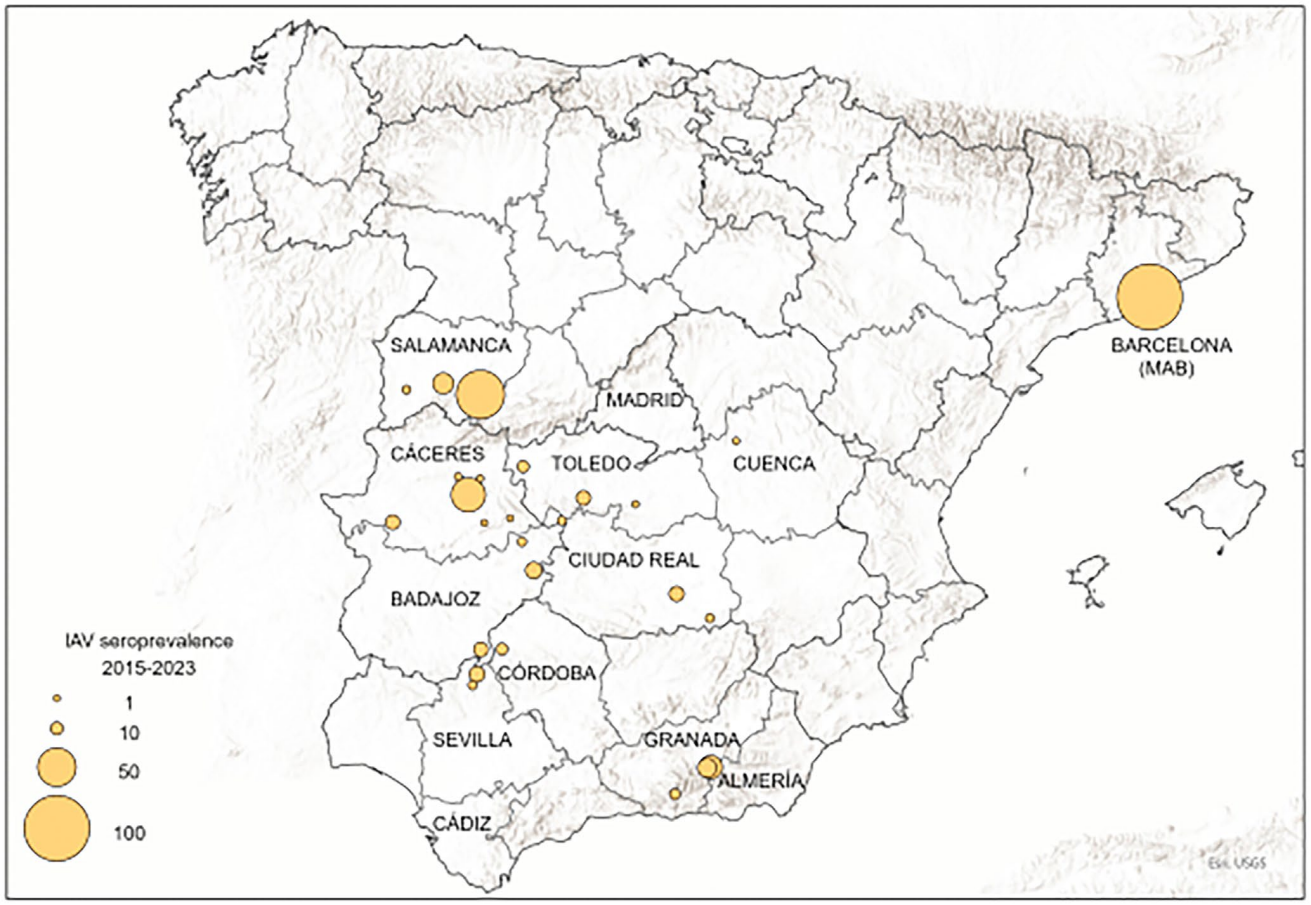
Geographical distribution of the influenza A seroprevalence. Circle size is proportional to seroprevalence in positive locations detected in Spain from 2015 to 2023. MAB: Metropolitan areas of Barcelona.

The spatial distribution analysis showed that the significantly highest seroprevalence with 95% central confidence interval was identified in the province of Salamanca (17%), followed by Badajoz (10%) and Toledo (9%) (Tables 4 and S2). The highest seropositivity (91.3%) was found in the samples collected in the MAB followed by Guijuelo (Salamanca) with 65% (Figure 5). The EAswH1 was the most frequently detected HA lineage in all provinces. Wild boar sera from Badajoz showed the major serotype diversity as they tested positive for all serotypes used in the HI assay, followed by Toledo that tested positive to 4 out of 5 (Table 4). There were sera that tested positive to two or more subtypes being the EAswH1/PDMswH1 combination the most common in all years/provinces followed by 2000s‐like H3/EAswH1 in Badajoz and Toledo in recent years (2022–2023) (Table S1). The MAB tested positive to two or more subtypes, with EAswH1/PDMswH1 or EAswH1/H3‐2000like/PDMswH1 as the most frequent HA combinations.

**TABLE 4:** Seroprevalence and HA lineage assignment of influenza A positive sera samples by province.

Province	Serologic test (NP ELISA) 2015–2023				Serotyping					
	Samples	IAV+	%	95% CI	EAswH1	PDMswH1	HUswH1	1970s‐like H3	2000s‐like H3	Undet
Almería	21	0								
Badajoz	255	25	10	6–13	17	11	1	2	8	1
Barcelona	23	21	91	80–100	13	12			4	6
Cáceres	625	13	2	1–3	8	7				4
Cádiz	129	0								
Ciudad Real	519	6	1	0–2	2					2
Córdoba	71	4	6	0–11	4					
Cuenca	54	1	2	0–5						1
Granada	109	5	5	1–9	3			1		1
Madrid	80	0								
Salamanca	143	24	17	11–23	19					5
Sevilla	207	14	7	3–10	14	2				1
Toledo	696	64	9	7–11	18	3	20		14	10

*Note:* Serological test: 1970s‐like H3, H3N2 with a haemagglutinin H3 from clade 1970.1; 2000s‐like H3, H3N1 subtype with a HA from clade 2000.3; EAswH1, H1N1 or H1N2 subtypes with a HA belonging to Eurasian avian like H1; HA, haemagglutinin; HUswH1, H1N2 subtype with a HA belonging to human seasonal‐like H1; IAV+, influenza A positive samples; NP ELISA, enzyme‐linked immunosorbent assay targeting IAV nucleoprotein; PDMswH1, H1N1 or H1N2 subtypes with a HA belonging to pandemic‐like H1.

**FIGURE 6:**
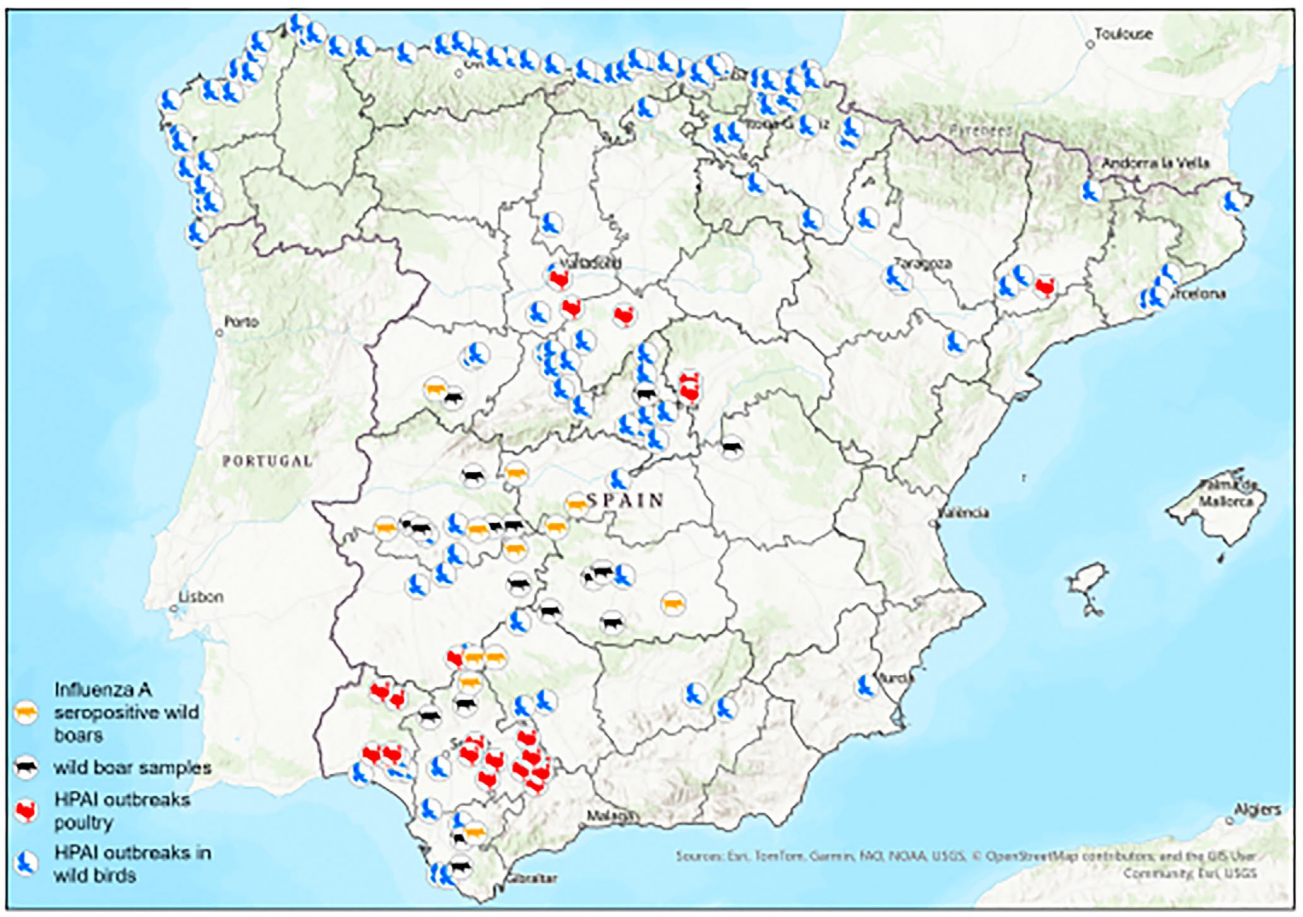
Map showing the seropositive/seronegative wild boar samples that collocate in areas with High Pathogenicity Avian Influenza (HPAI) H5N1 outbreaks in wild birds and poultry.

Among the 10 provinces where wild boar roaming areas overlapped with HPAI H5N1 outbreaks (2021–2023), 78 IAV‐positive sera were overall detected in 7 provinces: Toledo, Sevilla, Badajoz, Salamanca, Ciudad Real, Cáceres and Córdoba. None of these 78 sera tested positive against LPAI strains (H5N9, H7N1, H3N8) by HI. However, 21 of these samples tested positive for recombinant H5 haemagglutinin by ELISA. All the samples tested negative by HI using the HPAI H5N1 strain A/ *Anser anser* /Spain/88–3/2022 (Figure 6).

### 3.2 Virologic Assays

Only one sample out of 1643 was considered positive for IAV (Ct < 35) (Table 2). This sample, collected from Toledo province, was successfully isolated and propagated in MDCK cells, subtyped as H3N1 and registered under the name: A/wild boar/Spain/45560–1/2021. The clade assignment tool revealed that the haemagglutinin belonged to the novel human seasonal‐like H3 clade 2000.3, with a NA and an internal cassette from the Eurasian avian‐like lineage, so it was assigned to the previously described Spanish genotype 12 (Encinas et al. 2022). The full genome sequences are available in Genbank under the accession numbers: PP338805‐PP338812.1.

## 4 Discussion

It is largely known that wild boar is a suitable host for IAV (Jung et al. 2024) and interaction with wildlife, livestock and humans has been described (Cadenas‐Fernández et al. 2019; Conejero et al. 2024; Coz and Mathevet 2024; Johann et al. 2020; Triguero‐Ocaña et al. 2021). However, their role in IAV's epidemiology remains poorly understood. Although there is currently no official wild boar census in Spain, several studies have provided estimates of wild boar distribution and abundance based on models using hunting data and habitat suitability (Bosch et al. 2014; ENETWILD‐consortium et al. 2019; Ruiz‐Rodriguez et al. 2024), as also shown in the Figure S2. According to these studies, the wild boar population is widely distributed across Spain, with the highest densities estimated in the northeastern and south‐central regions. The latter, which is the main focus of our study, is particularly relevant for influenza transmission at the extensive livestock–wildlife interface due to overlapping areas with Iberian pigs (Ruiz‐Rodriguez et al. 2024) and the presence of swine influenza in this species (Encinas et al. 2022). This study provides one of the most comprehensive longitudinal serologic and virologic surveys of IAV exposure in wild boar populations in Spain, over nine consecutive years from 2015 to 2023. The findings confirm the continuous circulation of IAVs in wild boars and reveal significant temporal, geographical and subtype‐related patterns that contribute to a better understanding of IAV ecology at the wildlife‐livestock‐human interface.

The overall seroprevalence of 6% aligns with previous studies conducted in European wild boars, such as in northern Italy from 2007 to 2014 (De Marco et al. 2022), but our extended surveillance period allows for the observation of longer‐term epidemiological trends. Comparing inter‐annual seroprevalence, two different periods can be distinguished, from 2015 to 2018 where no statistically significant changes in seroprevalence were detected, and from 2020 to 2023, when statistically significant changes in seroprevalence were detected. The mean seroprevalence in the first period was 8.93%, lower than the 13.87% obtained between 2002 and 2010 in southern Spain (Cano‐Manuel et al. 2014) but similar to that obtained between 2011 and 2013 in wild boar populations from mid‐western Spain (Risco et al. 2015). The increase in geographical range and consistent sampling intensity from 2019 probably helped to detect the statistically significant increase from 2020 onwards (from 1.48% to 13.87%) after the absence of seropositivity in 2019 (Table 2). However, even the years with lower sampling intensity allowed us to detect the trend and shift in the variety of IAV strains circulating in the wild boar population (Table 3), thus confirming the usefulness of this species as a sentinel for diseases and pathogens circulating in the environment (Barroso et al. 2024).

Wild boar sera from Salamanca and Badajoz showed the significantly highest seroprevalence (17% and 10%, respectively) at the province level. This may be related to intraspecies transmission from Iberian pigs to wild boar, given that these provinces are principal producers of Iberian black pigs (Ministerio de Agricultura, Pesca y Alimentación 2015) (Table S3). The high seropositivity (91%) detected in the MAB raises concerns about IAV transmission risk in human‐wildlife interface zones.

Sera subtyping analysis confirmed the dominance of the EAswH1 lineage in the spatio‐temporal frame, consistent with its widespread circulation in European swine populations (Klivleyeva et al. 2024). The detection of the PDMswH1 lineage and the disappearance of the HUswH1 lineage after 2018 suggest dynamic shifts in viral reservoirs or reduced circulation of clade 1B strains. The emergence and sustained presence of the 2000s‐like lineage, a novel human‐seasonal H3 introduced into the European swine population in the 2000s (Anderson et al. 2021), from 2017 onwards, indicates a potential reverse zoonotic event and subsequent adaptation to suid hosts.

The only isolate achieved during this nine‐year sampling interval, A/wild boar/Spain/45560–1/2021 (H3N1), from Oropesa (Toledo), carries a human seasonal‐like H3 clade 2000.3. Genotyping of whole genome sequences assigned it to G12, a genotype previously isolated in white pigs in Toledo province in 2019 (Encinas et al. 2022). There was a 9% of IAV seroprevalence in this province, which also presented a wide variety of subtypes and serotypes, suggesting unseen transmission pathways, maybe from white pig to wild boar. Continuous monitoring of IAVs in wild boar populations is necessary globally to understand these threats and prevent viral transmission from wild boar to livestock and humans.

During our long‐term IAV serological surveillance study, HPAI outbreaks caused by the H5N1 clade 2.3.4.4.b had been reported during active and passive surveillance campaigns in Spain (Ministerio de Agricultura, Pesca y Alimentación 2024a). Coexistence between wild birds and wild boars is common, especially in wetlands (Barasona et al. 2021), and wild boars, as omnivorous animals, usually feed on dead animals, being birds up to 17.3% of their diet (Oja et al. 2017). The lack of serological evidence of wild boar contact with the HPAI A/ *Anser anser* /Spain/88–3/2022 (H5N1, 2.3.4.4.b) strain circulating in sympatric wild birds is comparable to previous reports on IAV transmission from ducks to wild boars in southern France wetlands (Vittecoq et al. 2012) or in wild pigs in southern China (Luo et al. 2013) and in Japan (Fujimoto et al. 2019). Nevertheless, this contrasts with serological evidence of H5N8 infection in wild boars detected by seroneutralisation in Germany in 2017 (Schülein et al. 2021), although results could not be confirmed by HI, and with serologically positive asymptomatic free‐ranging domestic pigs reported in Italy after direct contact with infected poultry (Rosone et al. 2023). IAV are rapidly mutating viruses (Shao et al. 2017) that can easily adapt to swine species (Rajao et al. 2019), where IAV reassortment of avian and mammalian IAV strains occurs (Brown 2000; Harder et al. 2013; Wright et al. 1992). Therefore, targeted surveillance of IAV in wild boar as a key indicator species of integrated wildlife monitoring (Barroso et al. 2024) is essential to forecast the potential of cross‐species transmission and consequent epidemics in new host species.

The increasing and expanding wild boar populations pose a management and health risk concern for the environment, wildlife, livestock and humans (Barasona et al. 2021; Fulgione and Buglione 2022; Meng et al. 2009). This has led to the research, development and implementation of specific management strategies (Escobar‐González et al. 2024; Ministerio de Agricultura, Pesca y Alimentación 2024b) and the inclusion of the species in wildlife health surveillance plans (Lawson et al. 2021; Ministerio de Agricultura, Pesca y Alimentación 2025b) in order to decrease the risk of wild boar transmission of diseases. This study reinforces the value of wild boar as a sentinel species for infectious disease monitoring, including Influenza A virus (Barroso et al. 2023). Longitudinal surveillance revealed temporal and spatial variability in seroprevalence and circulating subtypes, with notable differences between rural and peri‐urban areas. Although the H5N1 strain circulating in wild birds during the 2022–2023 outbreak was not detected in wild boars, high IAV seropositivity and subtype diversity were found in synurbic populations. Urban and peri‐urban environments, where humans and synurbic wildlife increasingly coexist (Ruiz‐Ponsell et al. 2024), represent critical interfaces for cross‐species transmission. The continued circulation of avian influenza viruses in such settings increases the likelihood of spillover events and potential viral adaptation to mammals, including wild boars and humans (Van Leeuw et al. 2025). These findings underscore the importance of targeted surveillance in anthropized environments, where conditions may favour viral reassortment and emergence.

## Author Contributions

**Paloma Encinas:** conceptualisation, investigation, methodology, visualisation, resources, writing – original draft preparation, writing – review and editing. **Aitor Nogales:** methodology, resources, writing – review and editing. **Estela Escribano‐Romero:** data curation, visualisation, writing – review and editing. **M. Ángeles Martín del Burgo:** investigation, methodology, visualisation, resources. **Jorge Ramón López‐Olvera:** conceptualization, resources, data curation, writing – review and editing. **José Enrique Granados:** conceptualisation, resources, data curation, writing – review and editing. **Gregorio Mentaberre:** resources, data curation, writing – review and editing. **Adolfo García‐Sastre:** funding acquisition, writing – review and editing. **Gustavo del Real:** conceptualisation, funding acquisition, visualisation, project administration, supervision, writing – review and editing.

## Funding

Research in G.R. and A.G.‐S. laboratories on influenza is partially funded by the Centre for Research on Influenza Pathogenesis and Transmission (CRIPT), one of the National Institute of Allergy and Infectious Diseases (NIAID) funded Centres of Excellence for Influenza Research and Response (CEIRR; contract #75N93021C00014). Wild boar sampling in the Metropolitan Area of Barcelona benefitted from the contracts 13/051, 15/0174, 16/0243 and 18/0243‐00PR‐01 funded by the Ajuntament de Barcelona, and the Research Grant PID2020‐115046GB‐I00 Ecología, salud pública y gestión del jabalí urbano, funded by the Spanish Ministerio de Ciencia e Innovación.

## Conflicts of Interest

The A.G.‐S. laboratory has received research support from Avimex, Dynavax, Pharmamar, 7Hills Pharma, ImmunityBio and Accurius, outside of the reported work. A.G.‐S. has consulting agreements for the following companies involving cash and/or stock: Castlevax, Amovir, Vivaldi Biosciences, Contrafect, 7Hills Pharma, Avimex, Pagoda, Accurius, Esperovax, Applied Biological Laboratories, Pharmamar, CureLab Oncology, CureLab Veterinary, Synairgen, Paratus, Pfizer, Virofend and Prosetta, outside of the reported work. A.G.‐S. has been an invited speaker in meeting events organised by Seqirus, Janssen, Abbott, Astrazeneca and Novavax. A.G.‐S. is inventor on patents and patent applications on the use of antivirals and vaccines for the treatment and prevention of virus infections and cancer, owned by the Icahn School of Medicine at Mount Sinai, New York, outside of the reported work. The other authors declare no conflicts of interest.

## Supporting information


**Data S1:** zph70040‐sup‐0001‐Supinfo.docx.

## Data Availability

The data that support the findings of this study are available from the corresponding author upon reasonable request. Supporting Information for this article is available in the supplementary section, containing supporting data that complement the findings presented.
