# Supplementary material for: Longitudinal Surveillance of Influenza A Virus Exposure in Wild Boars ( Sus scrofa ) in Spain (2015–2023): Serologic and Virologic Evidence of Subtype Infections and H5N1 Spillover Risk
Source: Zoonoses Public Health. 2026 Feb 10;73(3):191–204. doi: 10.1111/zph.70040 (PMC13053611; doi:10.1111/zph.70040)
Supplement: Supplementary file 1 — Data S1: zph70040‐sup‐0001‐Supinfo.docx. [file ZPH-73-191-s001.docx]

**SUPPORTING INFORMATION**


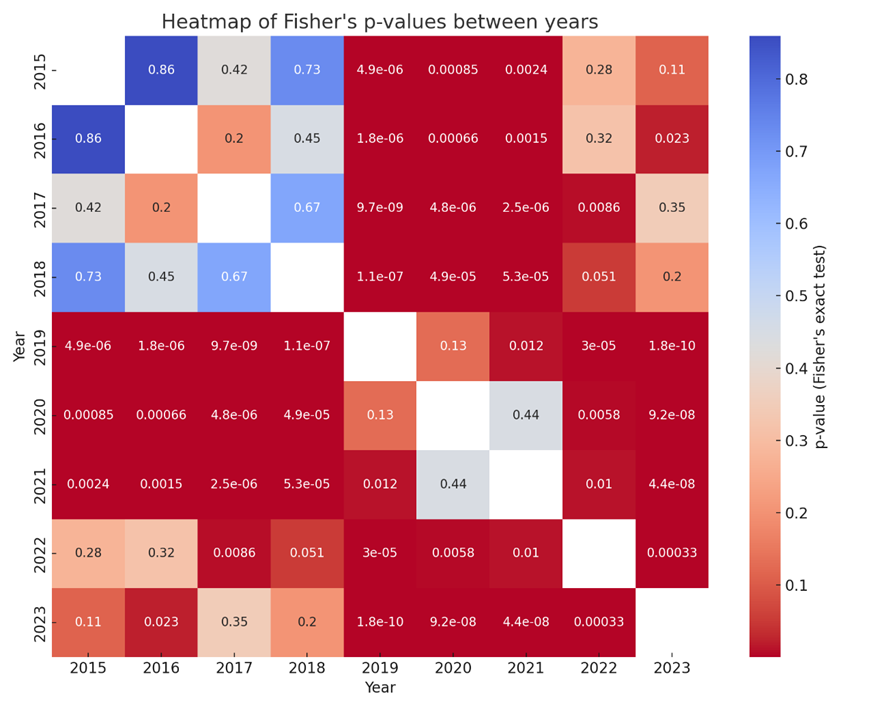


**Supplementary figure S1**: Fisher test results contrasting seroprevalence among years.


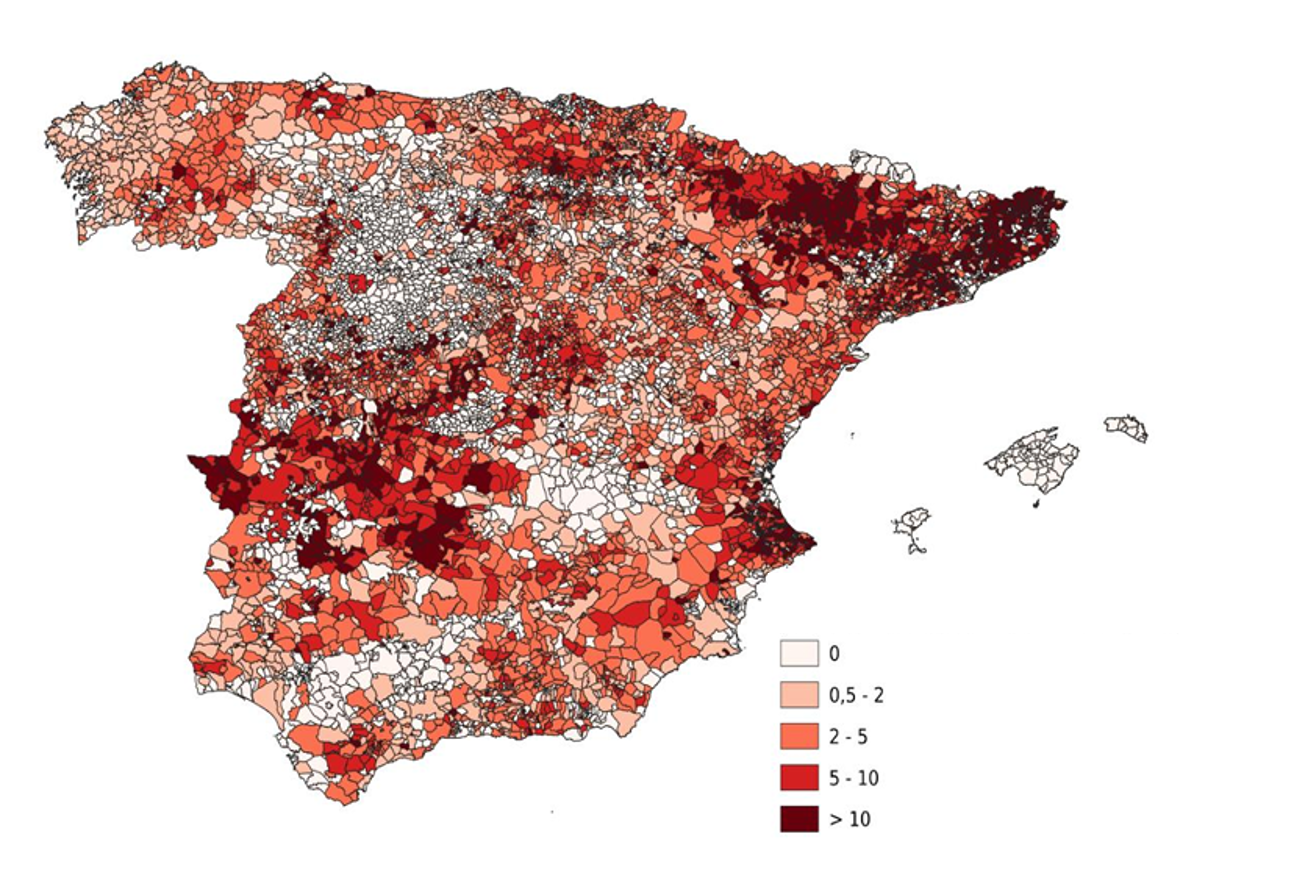


**Supplementary figure S2**: Estimated wild boar density. Number of wild boars/suitable surface (km2). Source: Ministry of Agriculture. Fisheries and Food of Spain (https://www.mapa.gob.es/dam/mapa/contenido/ganaderia/temas/produccion-y-mercados-ganaderos/caza/planes-y-estrategias/plannacionaldecontroldejabalieses.pdf)

**Table S1.** Distribution of influenza A positive wild boar sera samples by year, province and haemagglutinin lineages.

| **YEAR**  **PROVINCE**  **HA Lineage** | **IAV+ sera** | **1970s- like H3** | **2000s-like H3** | **HUswH1** | **EAswH1** | **PDMswH1** | **Undet** |
| --- | --- | --- | --- | --- | --- | --- | --- |
| **2015** | **12** |  |  | **9** | **3** | **3** |  |
| **CÁCERES** | **3** |  |  |  | **3** | **3** |  |
| EAswH1/PDM | 3 |  |  |  | 3 | 3 |  |
| **TOLEDO** | **9** |  |  | **9** |  |  |  |
| HUswH1 | 9 |  |  | 9 |  |  |  |
| **2016** | **24** | **2** |  | **11** | **11** | **9** | **3** |
| **BADAJOZ** | **12** | **2** |  | **1** | **9** | **9** | **1** |
| EAswH1 | 2 |  |  |  | 2 |  |  |
| EAswH1/PDM | 4 |  |  |  | 4 | 4 |  |
| H3-1970like/EAswH1/PDM | 2 | 2 |  |  | 2 | 2 |  |
| HUswH1/EAswH1/PDM | 1 |  |  | 1 | 1 | 1 |  |
| PDM | 2 |  |  |  |  | 2 |  |
| Undet | 1 |  |  |  |  |  | 1 |
| **CIUDAD REAL** | **1** |  |  |  |  |  | **1** |
| Undet | 1 |  |  |  |  |  | 1 |
| **CUENCA** | **1** |  |  |  |  |  | **1** |
| Undet | 1 |  |  |  |  |  | 1 |
| **TOLEDO** | **10** |  |  | **10** | **2** |  |  |
| HUswH1 | 8 |  |  | 8 |  |  |  |
| HUswH1/EAswH1 | 2 |  |  | 2 | 2 |  |  |
| **2017** | **30** | **1** | **4** |  | **19** | **15** | **7** |
| **BADAJOZ** | **3** |  |  |  | **3** | **1** |  |
| EAswH1 | 2 |  |  |  | 2 |  |  |
| EAswH1/PDM | 1 |  |  |  | 1 | 1 |  |
| **BARCELONA** | **21** |  | **4** |  | **13** | **12** | **6** |
| EAswH1 | 1 |  |  |  | 1 |  |  |
| EAswH1/PDM | 11 |  |  |  | 11 | 11 |  |
| H3-2000like | 3 |  | 3 |  |  |  |  |
| H3-2000like/EAswH1/PDM | 1 |  | 1 |  | 1 | 1 |  |
| Undet | 5 |  |  |  |  |  | 6 |
| **CÁCERES** | **2** |  |  |  |  | **2** |  |
| PDM | 2 |  |  |  |  | 2 |  |
| **GRANADA** | **4** | **1** |  |  | **3** |  | **1** |
| EAswH1 | 2 |  |  |  | 2 |  |  |
| H3-1970like/EAswH1 | 1 | 1 |  |  | 1 |  |  |
| Undet | 1 |  |  |  |  |  | 1 |
| **2018** | **25** |  |  | **1** | **19** | **3** | **7** |
| **CÁCERES** | **4** |  |  |  | **3** | **2** | **2** |
| EAswH1/PDM | 2 |  |  |  | 2 | 2 |  |
| Undet | 2 |  |  |  | 1 |  | 2 |
| **SALAMANCA** | **15** |  |  |  | **12** |  | **3** |
| EAswH1 | 12 |  |  |  | 12 |  |  |
| Undet | 3 |  |  |  |  |  | 3 |
| **SEVILLA** | **3** |  |  |  | **3** |  | **1** |
| EAswH1 | 2 |  |  |  | 2 |  |  |
| Undet | 1 |  |  |  | 1 |  | 1 |
| **TOLEDO** | **3** |  |  | **1** | **1** | **1** | **1** |
| EAswH1/PDM | 1 |  |  |  | 1 | 1 |  |
| HUswH1 | 1 |  |  | 1 |  |  |  |
| Undet | 1 |  |  |  |  |  | 1 |
| **2020** | **3** |  |  |  | **3** |  |  |
| **TOLEDO** | **3** |  |  |  | **3** |  |  |
| EAswH1 | 3 |  |  |  | 3 |  |  |
| **2021** | **11** |  | **3** |  | **5** |  | **3** |
| **CÁCERES** | **2** |  |  |  | **1** |  | **1** |
| EAswH1 | 1 |  |  |  | 1 |  |  |
| Undet | 1 |  |  |  |  |  | 1 |
| **CIUDAD REAL** | **3** |  |  |  | **2** |  | **1** |
| EAswH1 | 2 |  |  |  | 2 |  |  |
| Undet | 1 |  |  |  |  |  | 1 |
| **TOLEDO** | **6** |  | **3** |  | **2** |  | **1** |
| EAswH1 | 2 |  |  |  | 2 |  |  |
| H3-2000like | 3 |  | 3 |  |  |  |  |
| Undet | 1 |  |  |  |  |  | 1 |
| **2022** | **32** |  | **6** |  | **17** | **1** | **9** |
| **BADAJOZ** | **1** |  |  |  | **1** |  |  |
| EAswH1 | 1 |  |  |  | 1 |  |  |
| **CÁCERES** | **1** |  |  |  |  |  | **1** |
| Undet | 1 |  |  |  |  |  | 1 |
| **SALAMANCA** | **9** |  |  |  | **7** |  | **2** |
| EAswH1 | 7 |  |  |  | 7 |  |  |
| Undet | 2 |  |  |  |  |  | 2 |
| **SEVILLA** | **6** |  |  |  | **6** |  |  |
| EAswH1 | 6 |  |  |  | 6 |  |  |
| **TOLEDO** | **15** |  | **6** |  | **3** | **1** | **6** |
| EAswH1 | 2 |  |  |  | 2 |  |  |
| H3-2000like | 5 |  | 5 |  |  |  |  |
| H3-2000like/EAswH1 | 1 |  | 1 |  | 1 |  |  |
| PDM | 1 |  |  |  |  | 1 |  |
| Undet | 6 |  |  |  |  |  | 6 |
| **2023** | **33** |  | **13** |  | **21** | **4** | **2** |
| **BADAJOZ** | **9** |  | **8** |  | **4** | **1** |  |
| EAswH1 | 1 |  |  |  | 1 |  |  |
| H3-2000like | 5 |  | 5 |  |  |  |  |
| H3-2000like/EAswH1 | 2 |  | 2 |  | 2 |  |  |
| H3-2000like/EAswH1/PDM | 1 |  | 1 |  | 1 | 1 |  |
| **CÁCERES** | **1** |  |  |  | **1** |  |  |
| EAswH1 | 1 |  |  |  | 1 |  |  |
| **CÓRDOBA** | **4** |  |  |  | **4** |  |  |
| EAswH1 | 4 |  |  |  | 4 |  |  |
| **SEVILLA** | **5** |  |  |  | **5** | **2** |  |
| EAswH1 | 3 |  |  |  | 3 |  |  |
| EAswH1/PDM | 2 |  |  |  | 2 | 2 |  |
| **TOLEDO** | **14** |  | **5** |  | **7** | **1** | **2** |
| EAswH1 | 6 |  |  |  | 6 |  |  |
| EAswH1/PDM | 1 |  |  |  | 1 | 1 |  |
| H3-2000like | 5 |  | 5 |  |  |  |  |
| Undet | 2 |  |  |  |  |  | 2 |

**Table S2**: Fisher test results contrasting seroprevalence among provinces

|  |  |  |  |  |  |  |  |  |  |  |  |  |  |
| --- | --- | --- | --- | --- | --- | --- | --- | --- | --- | --- | --- | --- | --- |
|  | **ALMERÍA** | **BADAJOZ** | **BARCELONA** | **CÁCERES** | **CÁDIZ** | **C.REAL** | **CÓRDOBA** | **CUENCA** | **GRANADA** | **MADRID** | **SALAMANCA** | **SEVILLA** | **TOLEDO** |
| **ALMERÍA** |  | 0,234 | <0,0001**** | >0,9999 | <0,0001**** | >0,9999 | 0,5704 | >0,9999 | >0,9999 | >0,9999 | 0,0456* | 0,374 | 0,2451 |
|  | **BADAJOZ** |  | <0,0001**** | <0,0001**** | <0,0001**** | <0,0001**** | 0,3505 | 0,0599 | 0,1434 | 0,0011^**^ | 0,0556 | 0,3128 | 0,8018 |
|  |  | **BARCELONA** |  | <0,0001**** | <0,0001**** | <0,0001**** | <0,0001**** | <0,0001**** | <0,0001**** | <0,0001**** | <0,0001**** | <0,0001**** | <0,0001**** |
|  |  |  | **CÁCERES** |  | 0,1402 | 0,2531 | 0,0851 | >0,9999 | 0,1674 | 0,3809 | <0,0001**** | 0,0024^**^ | <0,0001**** |
|  |  |  |  | **CÁDIZ** |  | 0,6048 | 0,015^*^ | 0,2951 | 0,0192* | >0,9999 | <0,0001**** | <0,0001**** | <0,0001**** |
|  |  |  |  |  | **C.REAL** |  | 0,0231^*^ | 0,5018 | 0,0277* | >0,9999 | <0,0001**** | 0,0001*** | <0,0001**** |
|  |  |  |  |  |  | **CÓRDOBA** |  | 0,3885 | 0,7405 | 0,0467* | 0,0298^*^ | >0,9999 | 0,3869 |
|  |  |  |  |  |  |  | **CUENCA** |  | 0,6646 | 0,403 | 0,0033^**^ | 0,3204 | 0,076 |
|  |  |  |  |  |  |  |  | **GRANADA** |  | 0,0739 | 0,0025^**^ | 0,6195 | 0,1397 |
|  |  |  |  |  |  |  |  |  | **MADRID** |  | <0,0001^****^ | 0,013^*^ | 0,001^**^ |
|  |  |  |  |  |  |  |  |  |  | **SALAMANCA** |  | 0,0047** | 0,0104* |
|  |  |  |  |  |  |  |  |  |  |  | **SEVILLA** |  | 0,3247 |
|  |  |  |  |  |  |  |  |  |  |  |  | **TOLEDO** |  |

**Table S3** Influenza A seroprevalence and total number of pig by year/province

|  |  |  |  | **WHITE PIGS CENSUS** | | | | **IBERIAN PIGS CENSUS** | | | |
| --- | --- | --- | --- | --- | --- | --- | --- | --- | --- | --- | --- |
| **YEAR/PROVINCE** | **Total sera** | **Inf A + sera** | **%** | **MAY** | **NOVEMBER** | **TOTAL** | **%** | **MAY** | **NOVEMBER** | **TOTAL** | **%** |
| **2015** | 172 | 14 | 8 | 24.386.009 | 16.609 | 24.402.618 | 100% | 2.597.197 | 3.053.965 | 5.651.162 | 100 |
| CASTILLA LA MANCHA | 111 | 11 | 10 | 1.333.195 | 1.373 | 1.334.568 | 5% | 86.809 | 77.506 | 164.315 | 3% |
| TOLEDO | 90 | 11 | 12 | 833.776 | 803 | 834.579 | 3% | 72.034 | 68.229 | 140.263 | 2% |
| EXTREMADURA | 25 | 3 | 12 | 53.178 | 187 | 53.365 | 0% | 1.059.867 | 1.313.287 | 2.373.154 | 42% |
| CÁCERES | 17 | 3 | 18 | 9.607 | 41 | 9.648 | 0% | 117.253 | 96.634 | 213.887 | 4% |
| **2016** | 321 | 24 | 7 | 24.692.798 | 26.072.875 | 50.765.673 | 100% | 2.848.146 | 3.158.720 | 6.006.866 |  |
| CASTILLA LA MANCHA | 164 | 12 | 7 | 1.287.057 | 1.552.313 | 2.839.370 | 3% | 87.787 | 117.695 | 205.482 | 3% |
| CIUDAD REAL | 54 | 1 | 2 | 48.172 | 44.101 | 92.273 | 0% | 7.396 | 8.039 | 15.435 | 0% |
| CUENCA | 20 | 1 | 5 | 225.400 | 231.073 | 456.473 | 1% | 0 | 0 | 0 | 0% |
| TOLEDO | 85 | 10 | 12 | 767.758 | 1.019.518 | 1.787.276 | 4% | 80.390 | 109.656 | 190.046 | 3% |
| EXTREMADURA | 123 | 12 | 10 | 70.175 | 57.718 | 127.893 | 0% | 1.239.549 | 1.380.370 | 2.619.919 | 44% |
| BADAJOZ | 43 | 12 | 28 | 59.959 | 51.659 | 111.618 | 0% | 1.079.963 | 1.262.147 | 2.342.110 | 39% |
| **2017** | 291 | 31 | 11 | 25.835.187 | 26.864.589 | 52699776 | 100% | 2.831.535 | 3.106.767 | 5.938.302 |  |
| ANDALUCÍA | 121 | 5 | 4 | 1.649.498 | 1.718.151 | 3367649 | 6% | 721.338 | 893.531 | 1614869 | 27% |
| GRANADA | 101 | 5 | 5 | 0 | 202.213 | 202213 | 0% | 30.740 | 36.113 | 66853 | 1% |
| CATALUÑA | 23 | 21 | 91 | 7.719.446 | 7.754.295 | 15473741 | 29% | 0 | 0 |  |  |
| BARCELONA | 23 | 21 | 91 | 1.991.727 | 2.165.578 | 4157305 | 8% | 0 | 0 |  |  |
| EXTREMADURA | 102 | 5 | 5 | 42.004 | 32.872 | 74876 | 0% | 1.173.184 | 1.125.318 | 2298502 | 39% |
| BADAJOZ | 36 | 3 | 8 | 36.409 | 30.523 | 66932 | 0% | 1.068.288 | 996.503 | 2064791 | 35% |
| CÁCERES | 66 | 2 | 3 | 5.596 | 2.348 | 7944 | 0% | 104.896 | 128.814 | 233710 | 4% |
| **2018** | 254 | 26 | 10 | 27.089.597 | 27.517.008 | 54606605 | 100% | 3.051.661 | 3.287.091 | 6338752 |  |
| ANDALUCÍA | 23 | 3 | 13 | 1.684.212 | 1.654.100 | 3338312 | 6% | 752.733 | 970.564 | 1723297 | 27% |
| SEVILLA | 7 | 3 | 43 | 384.045 | 402.000 | 786045 | 1% | 213.445 | 268.845 | 482290 | 8% |
| CASTILLA LA MANCHA | 130 | 4 | 3 | 1.487.220 | 1.581.410 | 3068630 | 6% | 172.950 | 152.015 | 324965 | 5% |
| TOLEDO | 105 | 4 | 4 | 981.920 | 944.962 | 1926882 | 4% | 137.710 | 118.832 | 256542 | 4% |
| CASTILLA LEÓN | 35 | 15 | 43 | 3.155.138 | 3.190.081 | 6345219 | 12% | 856.824 | 930.669 | 1787493 | 28% |
| SALAMANCA | 35 | 15 | 43 | 75.127 | 67.347 | 142474 | 0% | 507.850 | 538.520 | 1046370 | 17% |
| EXTREMADURA | 47 | 4 | 9 | 52.789 | 47.023 | 99812 | 0% | 1.268.854 | 1.233.586 | 2502440 | 39% |
| CÁCERES | 45 | 4 | 9 | 10.056 | 5.390 | 15446 | 0% | 111.259 | 149.195 | 260454 | 4% |
| **2020** | 289 | 4 | 1 | 28.367.885 | 29.212.066 | 57579951 | 100% | 3.006.533 | 3.584.005 | 6590538 |  |
| CASTILLA LA MANCHA | 121 | 4 | 3 | 1.432.007 | 1.491.501 | 2923508 | 5% | 135.739 | 235.799 | 371538 | 6% |
| TOLEDO | 64 | 4 | 6 | 820.810 | 896.630 | 1717440 | 3% | 104.811 | 198.250 | 303061 | 5% |
| **2021** | 517 | 13 | 3 | 29.130.827 | 30.971.883 | 60102710 |  | 3.275.874 | 3.572.846 | 6848720 |  |
| CASTILLA LA MANCHA | 256 | 11 | 4 | 1.481.688 | 1.664.349 | 3146037 | 5% | 217.301 | 210.308 | 427609 | 6% |
| CIUDAD REAL | 121 | 5 | 4 | 18.734 | 23.970 | 42704 | 0% | 38.578 | 36.837 | 75415 | 1% |
| TOLEDO | 122 | 6 | 5 | 883.532 | 928.802 | 1812334 | 3% | 178.723 | 171.532 | 350255 | 5% |
| EXTREMADURA | 127 | 2 | 2 | 64.690 | 81.887 | 146577 | 0% | 1.315.821 | 1.444.760 | 2760581 | 40% |
| CÁCERES | 99 | 2 | 2 | 5.485 | 5.823 | 11308 | 0% | 138.788 | 145.722 | 284510 | 4% |
| **2022** | 608 | 33 | 5 | 29.512.054 | 30.676.785 | 60.188.839 |  | 3.040.542 | 3.396.595 | 6437137 |  |
| ANDALUCÍA | 119 | 6 | 5 | 1.822.759 | 1.865.665 | 3688424 | 6% | 702.708 | 835.207 | 1537915 | 24% |
| SEVILLA | 78 | 6 | 8 | 425.639 | 419.885 | 845524 | 1% | 180.456 | 228.840 | 409296 | 6% |
| CASTILLA LA MANCHA | 272 | 15 | 6 | 1.674.368 | 1.615.627 | 3289995 | 5% | 203.264 | 202.224 | 405488 | 6% |
| TOLEDO | 117 | 15 | 13 | 925.190 | 892.371 | 1817561 | 3% | 166.667 | 160.681 | 327348 | 5% |
| CASTILLA LEÓN | 26 | 9 | 35 | 3.446.291 | 3.309.173 | 6755464 | 11% | 1.055.914 | 1.122.103 | 2178017 | 34% |
| SALAMANCA | 26 | 9 | 35 | 52.262 | 83.559 | 135821 | 0% | 574.521 | 608.027 | 1182548 | 18% |
| EXTREMADURA | 179 | 3 | 2 | 44.502 | 46.392 | 90894 | 0% | 1.078.376 | 1.236.855 | 2315231 | 36% |
| BADAJOZ | 48 | 2 | 4 | 39.982 | 42.215 | 82197 | 0% | 976.015 | 1.092.661 | 2068676 | 32% |
| CÁCERES | 131 | 1 | 1 | 4.520 | 4.177 | 8697 | 0% | 102.361 | 144.194 | 246555 | 4% |
| **2023** | 240 | 32 | 13 | 30.814.559 | 30.189.917 | 61004476 |  | 3.043.953 | 3.613.123 | 6657076 |  |
| ANDALUCÍA | 58 | 9 | 16 | 1.862.365 | 1.898.744 | 3761109 | 6% | 615.576 | 734.201 | 1349777 | 20% |
| CÓRDOBA | 25 | 4 | 16 | 27.445 | 25.387 | 52832 | 0% | 181.659 | 232.324 | 413983 | 6% |
| SEVILLA | 20 | 5 | 25 | 401.032 | 406.137 | 807169 | 1% | 160.978 | 190.312 | 351290 | 5% |
| CASTILLA LA MANCHA | 86 | 14 | 16 | 1.686.920 | 1.669.479 | 3356399 | 6% | 181.053 | 169.380 | 350433 | 5% |
| TOLEDO | 39 | 14 | 36 | 962.906 | 961.320 | 1924226 | 3% | 147.626 | 137.143 | 284769 | 4% |
| EXTREMADURA | 76 | 9 | 12 | 63.873 | 61.067 | 124940 | 0% | 1.181.859 | 1.353.633 | 2535492 | 38% |
| BADAJOZ | 10 | 8 | 80 | 59.866 | 54.645 | 114511 | 0% | 1.049.825 | 1.181.442 | 2231267 | 34% |
| CÁCERES | 66 | 1 | 2 | 4.007 | 6.422 | 10429 | 0% | 132.034 | 172.191 | 304225 | 5% |
